# Supplementary material for: Impact of the COVID-19 pandemic on physical therapy practice in Saudi Arabia
Source: PLoS One. 2022 Dec 9;17(12):e0278785. doi: 10.1371/journal.pone.0278785 (PMC9733862; doi:10.1371/journal.pone.0278785)
Supplement: S1 File — (PDF) [file pone.0278785.s001.pdf]

Survey

Impact of the COVID-19 pandemic on the physical therapy practice in Saudi Arabia

Dear Physical Therapist,

The current COVID-19 pandemic has posed serious afflictions to the medical services worldwide. Due to the vast spread of the disease, the authorities had to impose precautionary measures including a complete lockdown. Since physical therapy service is a fundamental medical service, it is expected to be impacted by the imposed measures.

Therefore, our research team is conducting a study to assess the impact of the COVID-19 pandemic and the accompanying circumstances on the physical therapy practitioners and the rehabilitation services delivered in Saudi Arabia. We have designed a simple questionnaire comprised of 30 questions to assess different domains including how the pandemic impacted the physical therapy services, the response of the clinics to the pandemic, and the psychological distress physical therapists might have suffered because of the pandemic.

We are inviting you to fill out the questionnaire. Please be informed that your identity will be completely anonymous (no personal identifying information will be collected), and there are no consequences for refusing to participate. Your participation is voluntary.

This study has obtained ethical approval from the Institutional Review Board of the Prince Sultan Military College of Health Sciences (IRB No: IRB-2020-PT-032) If you have any further inquiries about the study, you may contact (Mohamed Alghamdi) by email mnghamdi@iau.edu.sa or phone (00966555811880).

By clicking the below link, you voluntarily agree to participate in this study, and give your consent to use your anonymous data for research purposes.

Sincerely,

Dr. Walaa Elsayed

Principal Investigator

<u>Questionnaire:</u> Impact of the COVID-19 pandemic on PT practice

<u>Section1: Demographic Data:</u>

- Age:
- Gender: Male ☐ Female ☐
- Nationality: Saudi ☐ Non Saudi ☐
- Do you have a valid physical therapy license? ☐ Yes | ☐ No
- Are you currently employed: ☐ Yes | ☐ No
- Work Region : Eastern ☐ Central ☐ Western ☐ Northern ☐ Southern ☐
- Professional rank : Assistant PT ☐ Junior PT ☐ Senior PT ☐ Assistant to Head of Department ☐ Head of Department ☐ consultant ☐
- Subspecialty: Musculoskeletal PT ☐ Pediatric PT ☐ Neurological PT ☐ Sports PT ☐ Cardiopulmonary PT ☐ Women Health PT ☐ Rehabilitation PT ☐ General PT ☐ other
- Education: Diploma ☐ Bachelor degree ☐ Master degree ☐ Doctoral degree ☐
- Years of experience: 0-2 ☐ 3-5 ☐ 6-10 ☐ 10-15 ☐ >15 ☐
- Employer: ☐ Governmental institution ☐ Private institution ☐ Non-governmental institution (Charity)

<u>Section 2 : impact on the PT practice</u>

1. Did you use tele-rehabilitation approach during the COVID-19 pandemic? ☐ Yes | ☐ No

2. If yes, how did you do it? Choose all that apply
   - Phone calls
   - Software applications
   - Video conferencing platform
   - Messages and emails

3. During the lockdown due to the COVID-19 pandemic, which of the following actions was taken by your clinic regarding the physical therapy services?
   - The clinic was completely shut down
   - The clinic was partially shut down (Only urgent, acute, post-surgical or inpatient cases managed)
   - Only Tele-rehabilitation was used to follow the patients at home
   - Combination of the previous actions

4. After the lockdown due to the COVID-19 pandemic was over, which of the following actions was taken by your clinic regarding the physical therapy services?
   - The clinic was completely shut down
   - The clinic was partially shut down (Only urgent, acute, post-surgical or inpatient cases managed)
   - Only Tele-rehabilitation was used to follow the patients at home
   - Combination of the previous actions

5. Have you taken a medical role outside your practice (assist triage, take vital signs, participate in COVID-19 awareness campaign)?

- always
- sometimes
- rarely
- never

6. During the COVID-19 pandemic, how frequently did you manage patients?

- none
- less than usual
- same as usual
- more than usual

7. Please rate your overall satisfaction with the physical therapy services you provided to your patients during the COVID-19 pandemic.

- dissatisfied
- partially satisfied
- satisfied
- completely satisfied

8. Please rate your overall satisfaction with how your department managed the physical therapy services during the COVID-19 pandemic.

- dissatisfied
- partially satisfied
- satisfied
- completely satisfied

9. Have you received education to adapt your physical therapy practice during the COVID-19 pandemic?

   ☐ Yes | ☐ No (yes: dropdown: online seminars, team meetings, in-person education, educational emails)

10. During the COVID-19 pandemic, have you been involved in the planning of service delivery at a strategic and operational level in your physical therapy department? ☐ Yes | ☐ No

11. During the COVID-19 pandemic, did your clinic respond appropriately and adapted the physical therapy services according to the guidelines and recommendations issued by scientific organizations? ☐ Yes | ☐ No

12. During the COVID-19 pandemic, which category of patients did you manage more?

- Acute cases
- Chronic cases

<u>Section 3: Psychological Distress:</u>

Over the last 2 weeks, how often have you been bothered by the following problems?

		Not at all	Several days	Over half the days	Nearly every day

1	Feeling nervous, anxious, or on edge	0	1	2	3
2	Not being able to stop or control worrying	0	1	2	3
3	Worrying too much about different things	0	1	2	3
4	Trouble relaxing	0	1	2	3
5	Being so restless that it's hard to sit still	0	1	2	3
6	Becoming easily annoyed or irritable	0	1	2	3
7	Feeling afraid as if something awful might happen	0	1	2	3

If you checked off any problems, how difficult have these made it for you to do your work, take care of things at home, or get along with other people?

Not difficult at all ___________
Somewhat difficult __________
Very difficult ______________
Extremely difficult __________

- Comments:

Do you have further comments you want to add?
