# Supplementary material for: Impact of the COVID-19 pandemic on physical therapy practice in Saudi Arabia
Source: PLoS One. 2022 Dec 9;17(12):e0278785. doi: 10.1371/journal.pone.0278785 (PMC9733862; doi:10.1371/journal.pone.0278785)
Supplement: Attachment — Submitted filename: comments.doc [file pone.0278785.s002.doc]

**Manuscript Number: ''PONE-D-21-28813'' entitled "Impact of the COVID-19 Pandemic on Physical Therapy Practice"**

Overall, the idea of research is very interesting. I appreciate your hard work on this interesting topic. I think the work is very nice and very detailed. However, there are some comments and suggestions.

**Revision Suggestions**

**Title**

- Brief and concise

**Abstract**

Line 36: "about 33% received **education** to adapt the PT practice"

You may use online courses, webinars instead

**Introduction**

- Well structured

**Materials and methods**

- Well structured

**Sampling**

- Line 109: (Physical therapists working in Saudi Arabia were invited to fill a…) how they were invited?

There are many factors that adversely affect psychological state and lead to anxiety:

- Did you consider whether the participant are native Saudi Arabian or from other nationalities?
- Lockdown, travel limitations, being affected with COVID-19 or losing beloved persons affect the psychological state. Did the authors considered these factors?

**Statistical analysis:**

- Well structured

**Results:** Satisfying

**Discussion**: Satisfying
