# Supplementary material for: Impact of the COVID-19 pandemic on physical therapy practice in Saudi Arabia
Source: PLoS One. 2022 Dec 9;17(12):e0278785. doi: 10.1371/journal.pone.0278785 (PMC9733862; doi:10.1371/journal.pone.0278785)
Supplement: Attachment — Submitted filename: Response to Reviewers docx.docx [file pone.0278785.s004.docx]

**Response to Reviewers’**

The authors would like to thank the editor and the reviewers for their time and their comments on our manuscript. Our response to the comments is posted beneath each original comment.

**Journal requirements:**

Title: please include the country where the study has been performed, i.e. 'Impact of the COVID-19 Pandemic on Physical Therapy Practice in Saudi Arabia'.

Response: The comment is addressed. The title has been updated.

**Reviewer #1**: the data submitted support the conclusion drawn from study. appropriate statistical approach has been adapted. the language of paper is understandable and does not contain errors. ethical approval was done for this study.

Response: the authors appreciate the reviewer’s comment.

**Reviewer #2:** Manuscript Number: ''PONE-D-21-28813'' entitled "Impact of the COVID-19 Pandemic on Physical Therapy Practice"
Overall, the idea of research is very interesting. I appreciate your hard work on this interesting topic. I think the work is very nice and very detailed. However, there are some comments and suggestions.
Revision Suggestions
Title
- Brief and concise
Abstract
Line 36: "about 33% received education to adapt the PT practice"
You may use online courses, webinars instead

Response: the authors appreciate the reviewer’s comment. The comment is addressed. Please check line 34

Introduction
- Well structured

Materials and methods
- Well structured

Sampling
- Line 109: (Physical therapists working in Saudi Arabia were invited to fill a…) how they were invited?

Response: the comment is addressed. The authors clarified this information at the revised manuscript version on line 107. “Physical therapists working in Saudi Arabia were invited through emails and social media to fill a web-based questionnaire”.

There are many factors that adversely affect psychological state and lead to anxiety:
- Did you consider whether the participant are native Saudi Arabian or from other nationalities?

Response: thank you for the meticulous observation and comment. We considered this information on the demographic data collected. We found that 92.7% of the respondents were Saudi.

- Lockdown, travel limitations, being affected with COVID-19 or losing beloved persons affect the psychological state. Did the authors considered these factors?

Response: we didn’t consider that in our survey since we used the GAD-7 score for testing anxiety which measures anxiety with no reflection for the causes behind it.

Statistical analysis:
- Well structured

Response: Thank you for your support

Results: Satisfying

Response: Thank you for your comment

Discussion: Satisfying

Response: Thank you for your comment
